# METTL3-Mediated m6A Modification Enhances the Function of Adipose-Derived Stem Cells Under Hypoxic Conditions Thereby Improving Fat Graft Retention

**DOI:** 10.1155/sci/5589397

**Published:** 2025-11-30

**Authors:** Yajie Guo, Mengmeng Hou, Jiawei Song, Han Peng, Shuai Liu, Jun Zhu, Qi Wang, Jipeng Li, Chenggang Yi, Huichen Li

**Affiliations:** ^1^Department of Digestive Surgery, Xijing Hospital, Fourth Military Medical University, Xi'an, China; ^2^Department of Graduate Studies, Xi'an Medical College, Xi'an, China; ^3^Department of Experimental Surgery, Xijing Hospital, Fourth Military Medical University, Xi'an, China; ^4^Department of Plastic and Reconstructive Surgery, Xijing Hospital, Fourth Military Medical University, Xi'an, China; ^5^Innovation Research Institute, Xijing Hospital, Fourth Military Medical University, Xi'an, China

**Keywords:** adipose-derived stem cells, anoxic preconditioning, fat transfer, m6A modification, METTL3, vascularization

## Abstract

**Background:**

Adipose-derived stem cells (ADSCs) have important application prospects in the field of regenerative medicine, such as adjuvant autologous fat transplantation (AFT), due to their multidirectional differentiation and immunomodulatory functions. However, functional limitation of ADSCs in hypoxic environments may affect their effectiveness in clinical applications. Hypoxic preconditioning is a potential strategy to improve the function of ADSCs by enhancing the antioxidant capacity and metabolic adaptations of the cells, but the optimal hypoxic conditions and the mechanism of action have not yet been clarified.

**Methods:**

ADSCs were extracted and pretreated with hypoxia in order to explore its effect on the function of ADSCs. The activity, apoptosis level, proliferation ability, and antioxidant capacity of ADSCs under normoxic and hypoxic conditions were evaluated using flow cytometry (FCM), live-dead cell fluorescence assay, and apoptosis flow assay. Further, the role of METTL3-mediated m6A modification in hypoxic preconditioning was explored by real-time fluorescence quantitative PCR, protein immunoblotting assay, and m6A modification level detection. Finally, the effect of hypoxic preconditioning of ADSCs on fat graft retention was verified by the mouse AFT model.

**Results:**

We found that the survival of ADSCs was not affected by 5% O_2_ pretreatment. Moreover, the cell viability and proliferation of ADSCs were enhanced after 24 h of anoxic preconditioning. Therefore, we determined that 5% O_2_ treatment for 24 h was the best hypoxic pretreatment condition for ADSCs, which enhanced the antioxidant capacity of ADSCs, reduced apoptosis. METTL3-mediated m6A modification played a critical role in hypoxic preconditioning to reduce apoptosis in ADSCs. It was verified in a mouse model that hypoxia preconditioning of ADSCs significantly improved adipose graft retention and promoted neovascularization.

**Conclusions:**

METTL3-mediated modification of m6A enhances the function of ADSCs under hypoxic conditions to improve adipose graft retention. These findings provide a new strategy and theoretical basis for improving the clinical outcome of fat grafting, as well as new molecular targets for future research.

## 1. Introduction

Mesenchymal stem cells (MSCs) have been found to possess properties such as multidirectional differentiation, immunomodulation and cytokine secretion [1–3]. Among them, adipose-derived stem cells (ADSCs) show typical characteristics of MSCs in vitro and are able to exert proangiogenic, antiapoptotic, antioxidative, and local stem cell activation effects on host cells or organs through immunomodulation [2, 4, 5]. It can be used in a wide range of regenerative medicine applications and is particularly suitable for assisted autologous fat grafting due to its adipose tissue-derived properties.

Autologous fat transplantation (AFT), also known as autologous fat filler or fat grafting, is a technique for correcting soft tissue contour deformities and repairing soft tissue defects. As a key technique in the field of plastic surgery, it has significant advantages, such as low immunogenicity, simple operation, low cost, and easy to obtain [6–9], has been widely used in the field of cosmetic and restorative reconstruction [10]. However, adipose tissue faces hypoxia and environmental acclimatization problems after transplantation, resulting in its susceptibility to liquefaction and necrosis [11, 12], enables up to 20%–80% fat resorption within 1 year after surgery [13]. This affects the long-term results of fat grafting and patient satisfaction [14, 15]. Previous studies have shown that ADSCs improve fat graft retention by promoting angiogenesis [16–21]. However, the hypoxic environment that ADSCs face after transplantation into the body leads to excessive accumulation of reactive oxygen species (ROS), triggering an imbalance between oxidative and antioxidant mechanisms [22], further leads to apoptosis [23]thereby reducing its effectiveness. In recent years, some studies have found that moderate hypoxia is a protective factor for ADSCs [24]. Thus anoxic pretreatment may be a potential strategy; however, the specific conditions regarding the optimal anoxic concentration and treatment time are currently unclear, and the underlying mechanisms need to be further explored.

Hypoxic preconditioning has been shown to activate multiple signaling pathways and enhance cellular antioxidant capacity and metabolic adaptations. In particular, N6-methyladenosine (m6A), as one of the most common chemical modifications in eukaryotic RNAs [25–27]. The role in cellular stress response has been gradually revealed in recent years. Among them, METTL3 as a key methyltransferase for m6A modification [28]. Its role in anoxic pretreatment has attracted much attention. Numerous studies have confirmed the correlation between hypoxia and m6A methylation [29–32]. Similarly, more and more evidence suggests that m6A is closely related to apoptosis, but most of the current studies on m6A and apoptosis remain in the phenotype, and the underlying mechanisms are still unclear. It has been reported in the literature that m6A modification levels are significantly up/downregulated in a variety of cells under hypoxic conditions; however, there have been few reports on the study of ADSCs. Therefore, we hypothesized that hypoxic preconditioning may affect the apoptotic process of ADSCs through m6A modification. In the present study, we systematically explored the role of hypoxic preconditioning in reducing m6A modification during apoptosis in ADSCs.

ADSCs play a crucial role in fat grafting, our study screened that 5% O_2_ treatment for 24 h is the optimal hypoxic preconditioning condition for ADSCs and improves grafted fat retention. In addition, METTL3-mediated m6A modification plays an important role in reducing the level of apoptosis in ADSCs by hypoxic preconditioning. We further validated the role of hypoxic preconditioning based on the mouse AFT model and found that hypoxic preconditioning of ADSCs was able to enhance the retention of AFT by promoting neovascularization. These findings provide a new strategy and theoretical basis for improving the clinical outcome of fat grafting.

## 2. Materials and Methods

### 2.1. ADSCs Extraction and Culture

Healthy and sterile human abdominal adipose tissue was obtained from the clinic and washed with saline or PBS to remove the swelling fluid. The adipose tissue was then mixed with 0.1% type I collagenase 1:1 and digested at 37°C for 45 min, then fresh complete medium was added to terminate the digestion and centrifuged for 5 min (1000 rpm/min). Afterward, the top layer was discarded, and only the bottom layer of cells was retained. The cells were resuspended with fresh complete medium, filtered through a cell strainer (100 µm), and all the cells were cultured in a CO_2_ cell culture incubator. Adipose tissue acquisition was reviewed and approved by the Ethics Committee of Xijing Hospital, and each donor signed a written informed consent form (Number KY20243548-1).

### 2.2. Flow Cytometric Identification of ADSCs

The prepared cell suspension was centrifuged for 5 min, the supernatant was discarded, washed with PBS, and resuspended. Add the flow-through antibody, incubate for 15 min under light, then wash with PBS and centrifuge for 5 min, then discard the supernatant and resuspend with 500 μL of PBS, and then test on the machine (the centrifugation speed is 1000 rpm/min).

### 2.3. Fluorescence Detection of Live and Dead Cells

The prepared cell suspension was centrifuged for 5 min and resuspended with complete medium, inoculated in 24-well cell culture plates (cell number 5 × 10^4^/well), and the subsequent experiments were carried out after anoxic pretreatment. Dilute the assay buffer with ddH2O, rinse the cells with 1×assay buffer to remove the residual esterase activity, and take the appropriate amount of Calcein-AM solution and PI solution to add into the 1×assay buffer to homogenize the staining working solution. Add 200 µL of staining working solution to each well, incubate at 37°C for 15 min, and wash with PBS 1–3 times, followed by fluorescence microscope to capture fluorescence images (analyze fluorescence using Image J software) (The centrifugation speed is 1000 rpm/min).

### 2.4. Apoptosis Flow-Through Assay

After anoxic pretreatment of cells, the culture medium was transferred to a centrifuge tube and set aside, the cells were washed with PBS and digested with trypsin, the digestion was terminated by adding cell culture medium, and the cell suspension was obtained by blowing with a pipette gun. Centrifuge for 5 min to obtain the cell precipitate; flow buffer resuspension of cells (cell concentration of 1 × 10^6^/mL), take 100 μL of cell suspension was transferred to the flow tube, the flow tube was added with Annexin V/FITC solution, mixing and incubated for 5 min away from the light, followed by adding 10 μL of 20 μg/mL PI solution and 400 μL PBS, and then on the machine for detection. (the centrifugation speed is 1000 rpm/min).

### 2.5. Apoptosis Fluorescence Assay

The cells were washed with PBS after anoxic preconditioning, fixed with 4% paraformaldehyde fixative for 30 min, washed with PBS for 2–3 times, added with PBS containing 0.3% Triton X-100, and incubated for 5 min. 50 μL of TUNEL assay solution was added and incubated for 60 min, protected from light, washed with PBS for two times, and then added with 1×DAPI staining solution and incubated for 5 min, protected from light; fluorescence images were acquired under a fluorescence microscope (fluorescence was analyzed using Image J software).

### 2.6. Cell Viability Assay

The prepared cell suspension was centrifuged for 5 min and resuspended with medium, inoculated into 96-well cell culture plates (cell number 3 × 10^3^/well), and then pretreated with anoxia for subsequent experiments. Add 10 μL of CCK-8 solution to each well, and continue to incubate for 0.5–4 h in a cell culture incubator at 37°C. Detect the absorbance (450 nm wavelength) by enzyme marker. Cell viability formula: [(As-Ab)/(Ac-Ab)] × 100% (As, absorbance of experimental wells; Ac, absorbance of control wells; Ab, absorbance of blank wells). The centrifugation speed was 1000 rpm/min.

### 2.7. Cell Proliferation Fluorescence Assay

The prepared cell suspension was centrifuged for 5 min and resuspended in complete medium, then inoculated into 24-well cell culture plates (cell number of 5 × 10^4^/well), and then pre-treated with hypoxia for subsequent experiments. Prepare 2×EdU working solution with fresh complete medium, add 200 μL of 2×EdU working solution and 200 μL of medium to each well, so that the final concentration of EdU is 50 μM. After mixing, incubate in a CO_2_ cell culture incubator for 6–7 h. Wash with PBS for three times, add cell fixative, and incubate for 15 min. Discard the fixative, wash with PBS for three times, add 1 mL of permeabilizing agent (0.3% PBS with Triton X-100) to each well. Add 1 mL of permeabilizer (0.3% Triton X-100 in PBS) to each well, incubate for 15 min at room temperature, discard the fixative, and wash with PBS three times (5 min/time). Add 200 μL of Click reaction mixture to each well, avoid light for 1–2 h, discard the click reaction solution, and wash with PBS for three times. Add 1×hoechst 33342 solution 200 μL to each well, incubate for 10 min at room temperature and avoid light, wash with PBS for 1–3 times, and then collect fluorescence images by fluorescence microscope (analyze the fluorescence with ImageJ software) (the centrifugation speed is 1000 rpm/min).

### 2.8. Reactive Oxygen Fluorescence Detection

The prepared cell suspension was centrifuged for 5 min and resuspended with complete medium, planted in 24-well cell culture plates (cell number of 5 × 10^4^/well), and then pre-treated with hypoxia for subsequent experiments. The DHE probe was diluted to a concentration of 10 μM using fresh serum-free medium, and 200 μL of DHE probe was added to each well. The cells were incubated at 37°C for 30 min, during which they were inverted and mixed several times, and the cells were washed by adding serum-free cell culture medium, and the fluorescent images were subsequently captured using a fluorescence microscope.

### 2.9. Real-Time Fluorescence Quantitative PCR Assay

Discard the cell culture medium and wash with PBS for 2–3 times, add 1 mL of Trizol and lyse at room temperature for 1–2 min, then transfer to a 1.5 mL EP tube by repeated blowing, add 200 μL of chloroform, mix well, let it stand at room temperature for 5 min, and then centrifuge at 4°C for 20 min (12,000 rpm/min). Then transfer the upper aqueous phase to a new EP tube; add 500 μL isopropanol, mix well and let stand for 10 min, then centrifuge at 4°C for 15 min (12,000 rpm/min), discard the supernatant, add precooled 75% ethanol, centrifuge at 4°C for 10 min (12,000 rpm/min), discard the supernatant, and dry it for 5–10 min. use DEPC water to solubilize RNA, and then test for the concentration of RNA. DEPC water was used to dissolve the RNA, and the RNA concentration was detected on the machine, and reverse transcription was carried out. Subsequently, real-time fluorescence quantitative PCR was performed.

### 2.10. Western Blotting

The cells were washed 2–3 times with precooled PBS at 4°C, and then 1 mL of strong lysis solution containing PMSF and phosphatase inhibitor was added, and the cells were lysed on ice for 30 min, and after lysis, the cells were transferred to 1.5 mL of EP and then centrifuged at 4°C for 30 min (12,000 rpm/min). The protein supernatant after centrifugation was collected, and the total protein was quantified using the BCA protein quantification kit. Prepare rapid electrophoresis solution. Electrophoresis was carried out with premade gel, the volume of protein sampling for each gel well was 20 μg, the volume of protein mark sampling was 5 μL, and the constant voltage was 150 V. Prepare rapid membrane transfer solution, the PVDF membrane was activated by methanol for 15 s, the sponge and its filter paper were wetted, and then placed in the order of negative pole-gel-membrane-positive pole, and the membrane was transferred for 15 min at a constant flow rate of 400 mA; at the end of the transfer, the PVDF membrane was placed in rapid closure solution, and the membrane was closed at room temperature. After membrane transfer, the PVDF membrane was placed in rapid sealing solution and sealed at room temperature for 20 min; next, wash with 1×TBST for 1–2 times; dilute primary antibody 1:1000, add primary antibody and incubate at 4°C overnight; recover primary antibody and wash with 1×TBST for three times, each time for 5 min; add HRP-labeled goat-anti-rabbit or anti-mouse secondary antibody, and incubate for 1 h on a shaking table at room temperature; prepare luminescence solution; immerse the membrane in luminescence solution for 5–10 s, and then test on the machine. The membrane was immersed in the luminescent solution for 5–10 s, and then detected on the machine.

### 2.11. m6A Modification Level Detection

After RNA extraction and quantification, follow the m6A RNA methylation quantification kit procedure, add 80 μL of binding solution to each well; add 2 μL of negative control, 2 μL of positive control, and 200 ng of RNA sample to the designated wells, and mix gently; add 50 μL of diluted capture buffer to each well; add 0.02, 0.2, 0.1, 0.4, and 1 ng of positive control to make a standard curve. 0.02, 0.2, 0.1, 0.4, and 1 ng of positive control; incubate at 37°C for 90 min, wash three times with 1× washing buffer; add 50 μL of diluted capture antibody to each well, incubate at room temperature for 60 min, wash three times with 1× washing buffer; add 50 μL of diluted detection antibody to each well, incubate at room temperature for 30 min, wash four times with 1× washing buffer; add 50 μL of diluted enhancer to each well, incubate at room temperature for 30 min, wash four times with 1× washing buffer; add 50 μL of dilution enhancer to each well, incubate at room temperature. Add 50 μL of dilution enhancer to each well, incubate for 30 min at room temperature, wash with 1× washing buffer for five times, add developing solution (100 μL/well), incubate for 10 min at room temperature, avoiding light, and monitor the color change of the positive control wells, and stop the reaction when the color changes to blue; add 100 μL of termination solution to each well, and test the absorbance value on the machine.

### 2.12. SiRNA Transfection

The siRNA-containing tubes were centrifuged instantly and lysed in DEPC water; cells were cultured in complete medium without antibiotics to ensure 50%–60% cell density at the time of transfection, and the complete medium was discarded prior to transfection, and fresh medium free of antibiotics and serum was added to prepare the siRNA and Lipofectamine 2000 mixture. After incubation for 20 min at room temperature, the siRNA and Lipofectamine 2000 mixture was added into six-well plates, the volume of each well was 500 μL, mixed well, and the cells were cultured in a CO_2_ cell culture incubator at 37°C for 5–6 h. The medium containing double antibiotics and serum was replaced, and the cells were cultured for 24–72 h. The knockdown efficiency of mRNA was detected by fluorescence quantification, and the level of protein expression was detected by protein immunoblotting. The protein expression level was detected by immunoblotting.

### 2.13. METTL3 Overexpression

Lentiviral vector (pCDH-CMV-METTL3-EF1*α*-Puro) was selected, and human-derived METTL3 CDS was cloned (without 3′ untranslated region [3′UTR] to avoid interference with potential shRNA). After validation by sequencing, HEK293T cells were cotransfected with the packaging plasmid (psPAX2/pMD2.G), and the viral supernatant was collected after 48–72 h. Viral supernatants were concentrated by ultracentrifugation, and titers were determined (target titer ≥1 × 10^8^ TU/mL). METTL3 knockdown (Si) cells were infected with METTL3-OE virus at MOI = 5–10, while 8 μg/mL polybrene was added to enhance the infection efficiency, and the medium was replaced with hygromycin-containing (OE screened) medium after 24 h. Overexpression efficiency was confirmed by qPCR and Western blot. The restoration of methyltransferase activity was further verified by m6A dot blot, and the functional rescue effect was assessed in combination with phenotyping experiments.

### 2.14. Fat Transfer

Male BALB/c nude mice aged 4–6 weeks were purchased from the Laboratory Animal Center of Air Force Medical University and were acclimatized and fed under SPF-grade experimental conditions for 1 week. The animals were divided into four groups: saline/normal oxygen ADSCs/hypoxia-pretreated ADSCs/hypoxia-pretreated ADSCs + METTL3 inhibitor (STM2457) group. First, anesthesia was administered with isoflurane, and the skin on the back of BALB/c nude mouse was sterilized with iodophor, then the adipose tissue mixture was prepared and implanted subcutaneously on the back of BALB/c nude mice. After 12 weeks of fat transplantation, BALB/c nude mice were executed by decapitation, the skin was incised with a scalpel, and the implanted adipose tissue was carefully peeled off with forceps. Subsequently, the specimens were fixed in a fat-specific fixative (at 4°C) after measurement of the volume and mass and were examined for fat retention, vascularization, and inflammatory infiltration. Note: Four BALB/c nude mice in each group, two transplantation sites were constructed in each group, the volume of fat implanted in each site was 200 μL, the amount of ADSCs was 1 × 10^6^, and the amount of METTL3 inhibitor STM2457 was 50 mg/kg.

### 2.15. Live Imaging

Male BALB/c nude mouse aged 4–6 weeks were purchased from the Laboratory Animal Center of Air Force Medical University and were acclimatized and fed under SPF-grade experimental conditions for 1 week. Experiments using OriCell GFP-labeled human ADSCs. Divided into three groups: normal oxygen ADSCs/hypoxia-pretreated ADSCs/hypoxia-pretreated ADSCs + METTL3 inhibitor (STM2457) group. First, anesthesia was administered with isoflurane, and the skin on the back of BALB/c nude mice was sterilized with iodophor, then the adipose tissue mixture was prepared and implanted subcutaneously on the back of BALB/c nude mice. Guillotine execution was performed on BALB/c nude mice at Days 0, 7, 14, and 28 of fat transplantation, respectively. The skin was incised with a scalpel during surgery, and the implanted adipose tissue was carefully peeled off with forceps. Subsequently, the samples were rinsed three times in precooled PBS, weighed after removing the surface liquid by suctioning with sterile filter paper, and laid flat in an imaging dish with a black background to avoid overlapping of tissues. An IVIS Spectrum imaging system (PerkinElmer) was used with the following parameter settings: excitation wavelength: 465–490 nm (GFP-specific excitation), emission wavelength: 515–575 nm, and exposure time: automatically optimized (usually 1–5 s). Note: Each group contained four BALB/c nude mice, and two transplantation sites were set up in each group, with 200 μL of adipose tissue implanted in each site, 1 × 10^6^ OriCell GFP-labeled human ADSCs were added, and the METTL3 inhibitor STM2457 was injected at a dose of 50 mg/kg.

### 2.16. Hematoxylin and Eosin Staining of Adipose Tissue

Baked slices; xylene dewaxing; alcohol rehydration; hematoxylin staining; 1% hydrochloric acid ethanol differentiation; 1% ammonia immersion; eosin staining; alcohol dehydration; xylene immersion; neutral resin sealing; microscopic observation; and photographs.

### 2.17. Masson Staining of Adipose Tissue

Baked slices; xylene dewaxing; alcohol rehydration; Weigert's iron hematoxylin staining; ethanol differentiation with 1% hydrochloric acid; rejuvenate red acidic magenta solution (Masson's stain A) staining; 1% phosphomolybdic acid solution differentiation; aniline blue or bright green solution (Masson's stain B) staining; 1% glacial acetic acid for a short period of time (1 min) for differentiation; alcohol dehydration; xylene immersion; neutral resin sealing; microscopic observation; and photographs.

### 2.18. Inflammatory Factor Analysis by ELISA

We use Thermo Fisher Scientific's IL-10 Uncoated ELISA Kit (BMS614) and IL-6 Uncoated ELISA (BMS603-2) test kits. Briefly, tissue samples were homogenized in RIPA lysis buffer containing protease inhibitors (1:10 w/v) and centrifuged at 12,000 × *g* for 15 min at 4°C. Supernatants were collected, and protein concentrations were determined by BCA assay for normalization. 96-well plates were coated with capture antibodies (1:100 dilution in PBS) overnight at 4°C. Nonspecific binding was blocked with 5% BSA for 2 h at RT. 100 μL of tissue lysates (diluted 1:5 in assay buffer) or standards were added in duplicate and incubated for 2 h at RT. After washing, biotinylated detection antibodies (1:250 dilution) were added for 1 h, followed by HRP-streptavidin (1:1000) for 30 min. TMB substrate was added for 15 min, stopped with 2N H_2_SO_4_. Absorbance was read at 450 nm using a microplate reader (BioTek ELx808).

### 2.19. Immunofluorescence Staining

The paraffin sections were placed on a 75°C baking machine for 4 –6 h, and then immersed in xylene I, II, and III for 15 min each to remove the wax and then washed with 100%, 95%, 85%, and 75% alcohol for 5 min each to rehydrate. Then, the samples were washed with PBS for 2–3 times, and antigen repair was carried out in acidic antigen repair solution for 20 min, then dried, treated with 3% hydrogen peroxide for 10 min, and washed with PBS three times, each time for 5 min, and labeled with a histochemical pen. Then 0.3% Triton X-100 was treated at room temperature for 10–15 min, washed with PBS for three times, each time for 5 min. Next, goat serum was blocked at 37°C for 30–60 min to remove the serum, and then the primary antibody was added directly (CD31 was diluted according to the concentration of 1:200), and then put in the wet box at 4°C overnight. After the overnight rewarming for 30 min, wash with PBS for three times, 5 min each time, then incubate with fluorescent secondary antibody (1:1000) for 1–2 h at room temperature, avoiding light, wash with PBS for three times, 5 min each time, add 1×DAPI staining solution and stain for 5–10 min, wash with PBS for three times, 5 min each time, and then seal the slides with antifluorescent quencher and collect the fluorescence images with a laser confocal microscope.

### 2.20. Bioinformatics Analysis

The prediction of m6A modification sites and secondary structure analysis of Bcl-2 mRNA were performed using the SRAMP database (http://www.cuilab.cn/sramp). To identify METTL3 binding sites and characterize the predominant m6A motifs within target transcripts, we utilized RMBase v2.0 (https://rna.sysu.edu.cn/rmbase/). Additionally, potential interaction regions between Bcl-2 mRNA and METTL3 protein were predicted through catRAPID omics v2.0 (http://service.tartaglialab.com/page/catrapid_omics2_group).

### 2.21. m6A-RNA and RNA Immunoprecipitation (RIP) Assays

For m6A-RNA immunoprecipitation (meRIP), we employed the RiboCluster Profiler RIP-Assay Kit (RN1001, MBL, Japan) following the manufacturer's instructions. Briefly, RNA samples were incubated with magnetic beads conjugated to m6A-specific antibody (ab286164, 5 μg; Abcam). After washing, bound RNAs were eluted and purified for subsequent RT-qPCR analysis of target gene expression.

For RIP assays, we used the same kit with magnetic beads conjugated to either METTL3 antibody (15073-1-AP, 5 μg; Proteintech) or IgG control. Immunoprecipitated RNAs were analyzed by RT-qPCR to quantify Bcl-2 mRNA enrichment levels.

### 2.22. Statistical Analyses

We used the unpaired Student's-*t* test to analyze statistical differences between the two groups of data and one-way analysis of variance (ANOVA) as well as Tukey's post hoc test to analyze differences between multiple groups of data. *p* < 0.05 was considered statistically significant.

## 3. Results

### 3.1. 5% O_2_ Treatment for 24 h is the Optimal Hypoxic Preconditioning Condition for ADSCs

ADSCs promote angiogenesis, prevent apoptosis, resist oxidative stress, and activate local stem cells [33], thus, it plays a key role in the process of tissue repair and regeneration. In this study, we obtained healthy and sterile human abdominal adipose tissue from the clinic, and after extracting primary ADSCs and culturing them for 48 h, a large number of pancake-shaped erythrocytes and spindle-shaped ADSCs were visible under the microscope (Supporting Information: Figure S1). Previous studies have shown that MSC are able to express CD90, CD105, and CD73, but not CD45, CD34, CD14, CD11b, CD79a, CD19, and HLA-DR [34]. To demonstrate the successful extraction of ADSCs, we analyzed the specific membrane surface markers of the extracted cells based on flow cytometry (FCM), which showed that the stem cell markers CD45 (1.8%), CD11b (1.3%), and CD34 (1.9%) were negative, while CD44 (99.0%), CD90 (98%), CD105 (98.6%), and CD29 (99.6%) were positive (Supporting Information: Figure S2A).

**Figure 1:**
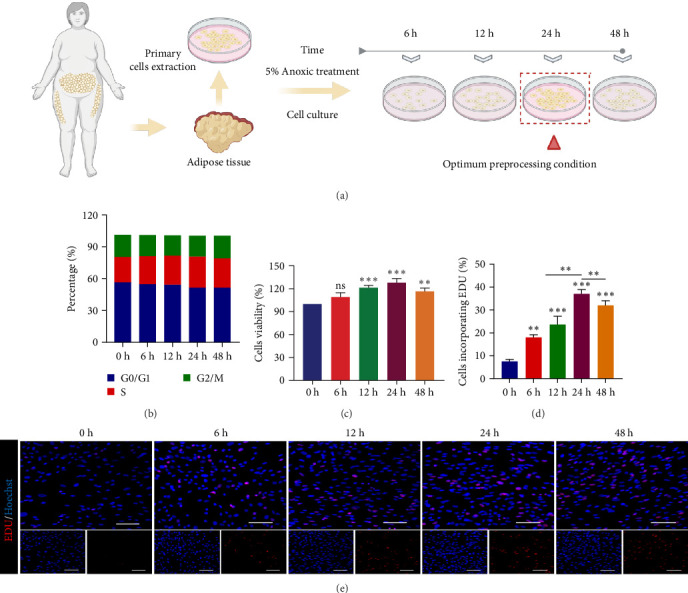
ADSCs cell cycle assay and cell proliferation capacity assay. (A) Flowchart for screening optimal conditions for anoxic preconditioning of ADSCs. (B) Cell cycle statistics. (C) Cell viability assay of ADSCs after hypoxic preconditioning. (D) EDU staining statistics of ADSCs after hypoxic pretreatment. (E) EDU staining of ADSCs after hypoxic preconditioning (scale bar, 100 μm). Data are presented as mean ± SD. Statistical significance: *⁣*^*∗*^*p* < 0.05, *⁣*^*∗∗*^*p* < 0.01, and *⁣*^*∗∗∗*^*p* < 0.001.

It is well known that the therapeutic efficacy of MSCs is severely reduced after cell transplantation due to exposure to extreme ischemic and hypoxic environments. Whereas it has been shown [35]that moderate hypoxia is a protective factor, an O_2_ concentration of 2%–7% can well mimic the in vivo environment of cells. Therefore, we set up different time gradients to determine the optimal time of hypoxic preconditioning by monitoring the status of the cells (Figure 1A), and the results showed that there was no significant change in the red fluorescence level of ADSCs after 6, 12, 24, and 48 h of treatment with 5% O_2_ compared to incubation in normoxic conditions (20%–21% O_2_, Supporting Information: Figure S2B), which suggests that the 5% O_2_ environment did not cause the ADSCs to die and their survival status was good. To further confirm the above results, we examined the apoptosis levels of ADSCs under 5% O_2_ conditions based on FCM and TUNEL fluorescence staining. The results showed that there was no significant change in the level of apoptosis in ADSCs after 6 (8.2%), 12 (8.1%), 24 (7%), and 48 h (7.3%) of treatment with 5% O_2_ compared to normoxic conditions (8.2%) (Supporting Information: Figure S2C). Similarly, TUNEL fluorescence staining revealed no significant difference in apoptotic cells between groups (Supporting Information: Figure S2D), which is consistent with the results of Calcein AM/PI cell viability level assay. Therefore, we can conclude that the survival of ADSCs is not affected by pretreatment with 5% O_2_.

In addition, we speculate that the cell cycle is likely to change in response to changes in the external environment. Therefore, we examined the cell cycle and detected a decrease in the proportion of G1-phase cells and an upregulation in the proportion of S-phase cells in ADSCs after 5% O_2_ pretreatment (Figure 1B), which suggests that our changes with the duration of hypoxic pretreatment may affect the proliferative capacity of ADSCs. Therefore, we further examined the changes in cell viability levels and cell proliferation changes in ADSCs after hypoxic preconditioning. First we performed CCK-8 assay after 6, 12, 24, and 48 h of hypoxic preconditioning, and the results showed that ADSCs were significantly enhanced in preconditioned (5% O_2_) conditions, and the cell viability in the 24 h group was significantly different compared with that in the normoxic group (Figure 1C). Second, we examined ADSCs based on EDU cell proliferation kit and found that compared with the normoxic group, the proliferation ability of ADSCs was significantly increased with the prolongation of hypoxic preconditioning time. Among them, the 24 h group had the strongest cell proliferation ability, with a significant difference of *p*  < 0.01 compared with the rest of the groups (Figure 1E,D).

In summary, we found that the survival of ADSCs was not affected by 5% O_2_ pretreatment. Moreover, the cell viability and proliferation of ADSCs were enhanced after 24 h of anoxic preconditioning, so we found that 5% O_2_ treatment for 24 h was the best anoxic preconditioning condition for ADSCs.

### 3.2. Enhanced Antioxidant Capacity and Decreased Apoptosis Levels in ADSCs After Hypoxic Preconditioning

**Figure 2:**
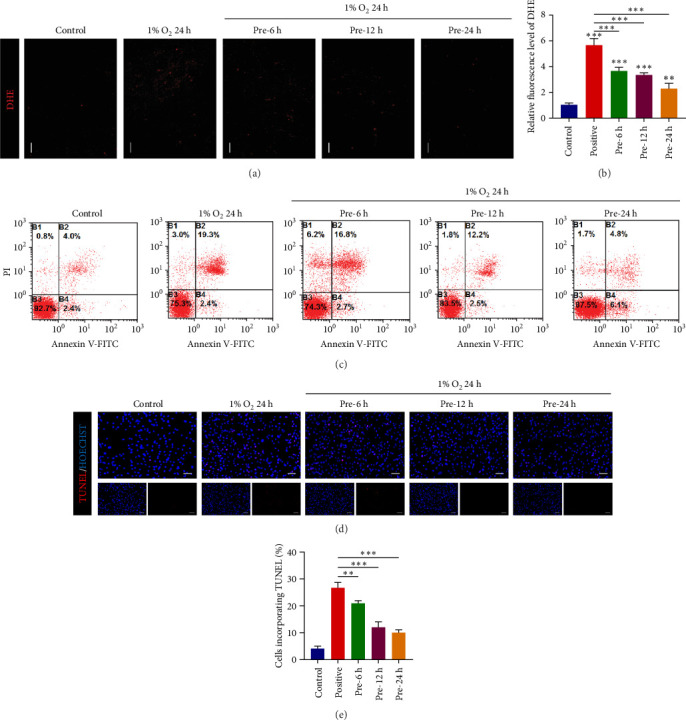
Detection of antioxidant capacity and apoptosis level of ADSCs after hypoxic preconditioning. (A) DHE fluorescence staining of ADSCs (scale bar, 100 μm). (B) DHE fluorescence intensity statistics of ADSCs. (C) Apoptosis flow-through assay after hypoxic pretreatment of ADSCs. (D) TUNEL fluorescence staining of ADSCs after hypoxic preconditioning (scale bar, 100 μm). (E) TUNEL fluorescence staining statistics. Data are presented as mean ± SD. Statistical significance: *⁣*^*∗*^*p* < 0.05, *⁣*^*∗∗*^*p* < 0.01, and *⁣*^*∗∗∗*^*p* < 0.001.

Previous studies have shown that pretreatment of MSCs by various factors such as cytokines, hypoxia, chemical agents, physical factors, and growth factors enhances the performance of MSCs [36]. We therefore examined the reactive oxygen levels of ADSCs after hypoxic preconditioning (5% O_2_, Figure 2A). Compared with the normoxic group (control group), the ROS levels of ADSCs were significantly increased (*p*  < 0.001) after 24 h of extreme hypoxia treatment with 1% O_2_ (positive control group), whereas the ROS levels of ADSCs were significantly decreased (*p*  < 0.001) after hypoxic preconditioning for 6, 12, and 24 h followed by extreme hypoxic stimulation compared to the positive control group (Figure 2B). This suggests that our pretreatment at 5% O_2_ enhances the antioxidant capacity of ADSCs and reduces the risk of oxidative damage in ADSCs due to extreme hypoxia.

**Figure 3:**
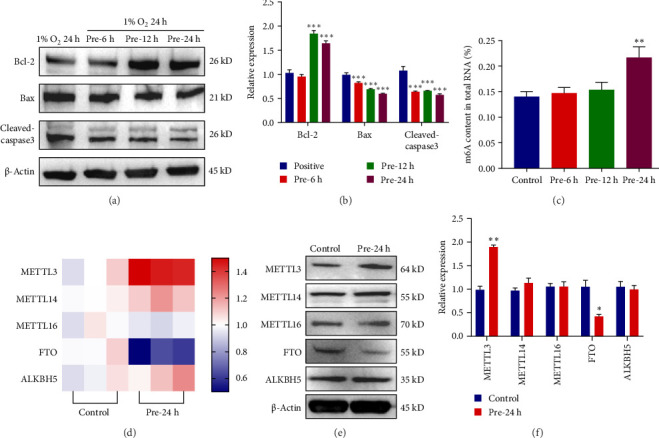
Expression of apoptosis-related molecules after hypoxic preconditioning of ADSCs and screening of key molecules affecting m6A modification levels. (A) Protein immunoblotting experiments of apoptosis-related molecules after hypoxic preconditioning of ADSCs. (B) Protein expression statistics of apoptosis-related molecules. (C) Detection of m6A content of total RNA in ADSCs after hypoxia pretreatment based on the m6A RNA Methylation Assay kit. (D) Real-time fluorescence quantitative PCR screening for key molecules affecting m6A modification levels. (E) Protein immunoblotting assay to screen the key molecules affecting the m6A modification level. (F) Statistics of protein immunoblotting assay results. Data are presented as mean ± SD. Statistical significance: *⁣*^*∗*^*p* < 0.05, *⁣*^*∗∗*^*p* < 0.01, and *⁣*^*∗∗∗*^*p* < 0.001.

Previous studies have shown [37] that a decrease in mitochondrial membrane potential during excessive cellular ROS production leads to an increase in the permeability of the inner mitochondrial membrane and the release of proteins, such as cytochrome C from the mitochondria. In contrast, cytochrome C binds to the apoptotic protein Apaf-1 to form apoptotic bodies, which activate cysteinyl asparagin-activating factor (caspase), ultimately triggering apoptosis. In the above study we learned that hypoxic preconditioning reduces ROS levels in ADSCs under extreme hypoxic conditions, and we hypothesized that apoptosis levels in ADSCs would be similarly affected. Therefore apoptosis of ADSCs was detected based on FCM and TUNEL fluorescence staining. The flow-through results showed a significant upregulation of apoptosis levels in ADSCs treated with extreme hypoxia (1% O_2_) for 24 h (21.7%) compared to the control group (6.4%), and a significant downregulation of apoptosis levels after hypoxic pretreatment for 6 h (19.5%), 12 h (14.7%), and 24 h (10.9%, Figure 2C). The results of TUNEL apoptosis assay were consistent with the above results (Figure 2D), in which the apoptosis level was the lowest at 24 h of hypoxic preconditioning, and there was a significant difference (*p*  < 0.05) compared with other groups (Figure 2E). In addition, we also examined the expression of related molecules affecting apoptosis (Figure 3A,B), among which Bcl-2 is an antiapoptotic protein, and we found that the expression level of Bcl-2 was upregulated after hypoxic preconditioning, and the expression level of the proapoptotic protein Bax was downregulated. Cleaved caspase-3 is a protein hydrolase closely related to apoptosis, it is the activated form of caspase-3, it can specifically shear a variety of proteins, leading to DNA cleavage and cell death, it is only found in apoptotic cells, and it can be used as a marker of apoptosis [38]. We found that the expression level of cleaved caspase 3 decreased after hypoxic preconditioning. Taking into account the above experimental results, we know that hypoxic preconditioning can reduce the apoptosis level of ADSCs under extreme hypoxic conditions, and we will explore the mechanism of this in the next section.

### 3.3. METTL3-Mediated m6A Modification Reduces Apoptosis Levels in ADSCs Under Conditions of Hypoxic Preconditioning

We found that the apoptosis level of ADSCs was significantly reduced after hypoxic preconditioning, and further investigation of the mechanism by which hypoxia affects apoptosis is of great significance for the treatment of ADSCs. Growing evidence suggests that m6A is closely associated with apoptosis [39]. Another study [40] showed that m6A levels were altered in a variety of cells under different stress conditions. Regarding the changes in the apoptosis level of ADSCs after hypoxic preconditioning, we hypothesized that m6A might play a key role in this process. Therefore, we further examined the m6A levels of ADSCs after hypoxic preconditioning and found that the m6A levels of ADSCs were significantly upregulated (*p* < 0.01) after 24 h of 5% O2 preconditioning (Figure 3C). Therefore, we next screened the key molecules affecting the changes of m6A levels in ADSCs by molecular biology experiments (Figure 3D, E), and found that the expression levels of methyltransferase METTL3 were significantly up-regulated in ADSCs after 24 h of hypoxic preconditioning (*p* < 0.01), and that of demethylase FTO were down-regulated (*p* < 0.05), while the expression levels of METTL14, METTL16, and ALKBH5 were not significantly changed (Figure 3F). Several studies have shown that METTL3 is a common molecule affecting m6A levels and is widely involved in physiopathological processes [41], so we hypothesized that METTL3 may play an important role in the changes of m6A levels in ADSCs affected by hypoxic preconditioning.

**Figure 4:**
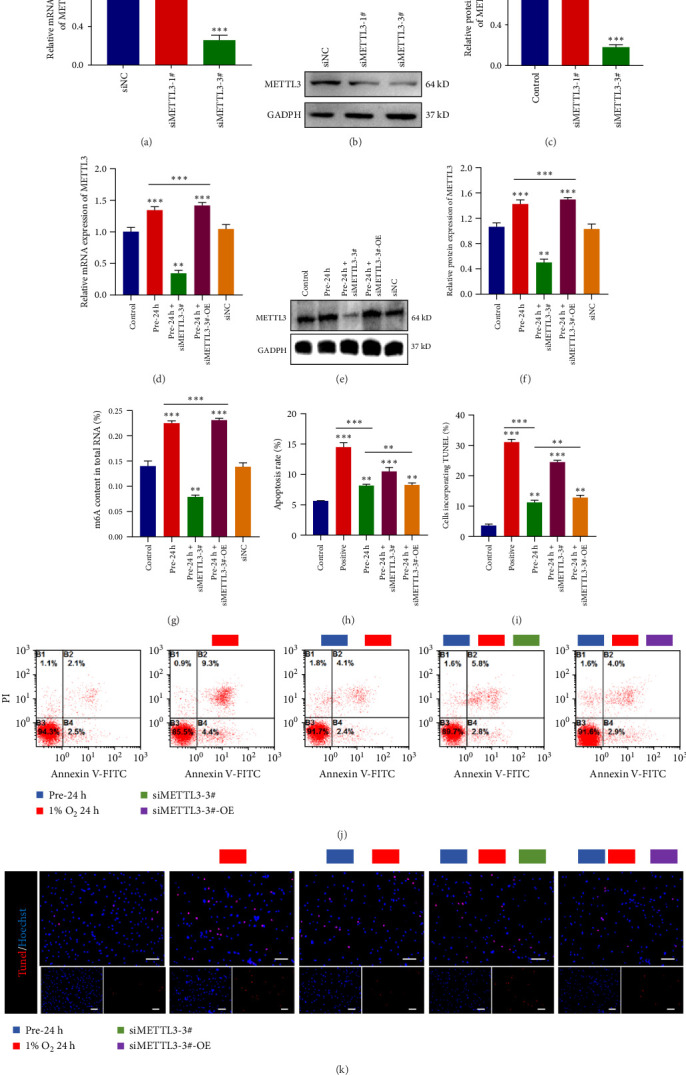
Validation of the role of METTL3 in changes in m6A levels in hypoxia-preconditioned ADSCs and their apoptotic levels. (A) Real-time fluorescence quantitative PCR assay to detect the mRNA expression level of METTL3. (B) Protein immunoblotting assay to detect the protein expression level of METTL3. (C) Statistics of protein immunoblotting results. (D) Real-time fluorescence quantitative PCR assay to detect the mRNA expression level of METTL3. (E) Protein immunoblotting assay to detect the protein expression level of METTL3. (F) Statistics of protein immunoblotting results. (G) Detection of ADSCs m6A level. (H) Statistics of flow assay for apoptosis. (I) Statistics of apoptosis TUNEL fluorescence staining. (J) Flow-through detection of apoptosis. (K) TUNEL fluorescence staining for apoptosis (scale bar, 100 μm). Data are presented as mean ± SD. Statistical significance: *⁣*^*∗*^*p*  < 0.05, *⁣*^*∗∗*^*p*  < 0.01, and *⁣*^*∗∗∗*^*p*  < 0.001.

Next, to verify whether METTL3 plays a critical role in the process of changing m6A modification levels in ADSCs. We used siRNA to knock down METTL3 expression in ADSCs, and the results showed that siMETTL3-3# had a knockdown efficiency of more than 60% (*p* < 0.001) for METTL3 in ADSCs (Figure 4A), and protein immunoblotting experiments also confirmed that siMETTL3-3# had a good knockdown efficiency (*p* < 0.001, Figure 4B,C). To exclude off-target effects and confirm the specific function of METTL3, we further constructed a METTL3 overexpression rescue system. Validated by qPCR and Western blot, the results showed that METTL3 expression in the overexpression group was restored to 1.4 ± 0.2-fold of that in the control group (*p* < 0.001, Figure 4D–F). overexpression of METTL3 was able to significantly reverse the decrease in m6A modification level caused by knockdown (*p* < 0.001, Figure 4G), which fully confirmed the central role of METTL3 in the regulation of m6A modification in ADSCs. the central role of METTL3 in the regulation of m6A modification in ADSCs. Based on this, we further explored the role of METTL3-mediated m6A modification in the reduction of apoptosis levels in ADSCs by hypoxic preconditioning. The results of apoptosis FCM assay showed that under hypoxic preconditioning conditions, the apoptosis level of ADSCs in the METTL3 knockdown group was significantly increased in extreme hypoxic environment, while METTL3 overexpression effectively reversed this phenomenon (Figure 4H,J). In addition, the results of TUNEL fluorescence staining experiments were consistent with the flow-through results (Figure 4I,K), further validating our findings. It was shown that METTL3 plays an important role in reducing the apoptotic level of ADSCs by hypoxic preconditioning through regulating m6A modification.

This study explored the mechanism by which m6A-modified hypoxic preconditioning reduces the level of apoptosis in ADSCs, elucidated the molecular mechanism of METTL3-mediated m6A modification in the apoptotic process of ADSCs, and provided targets of action to further improve the clinical therapeutic efficacy of ADSCs.

### 3.4. Hypoxia Preconditioning of ADSCs Promotes Retention of Fat Grafts

**Figure 5:**
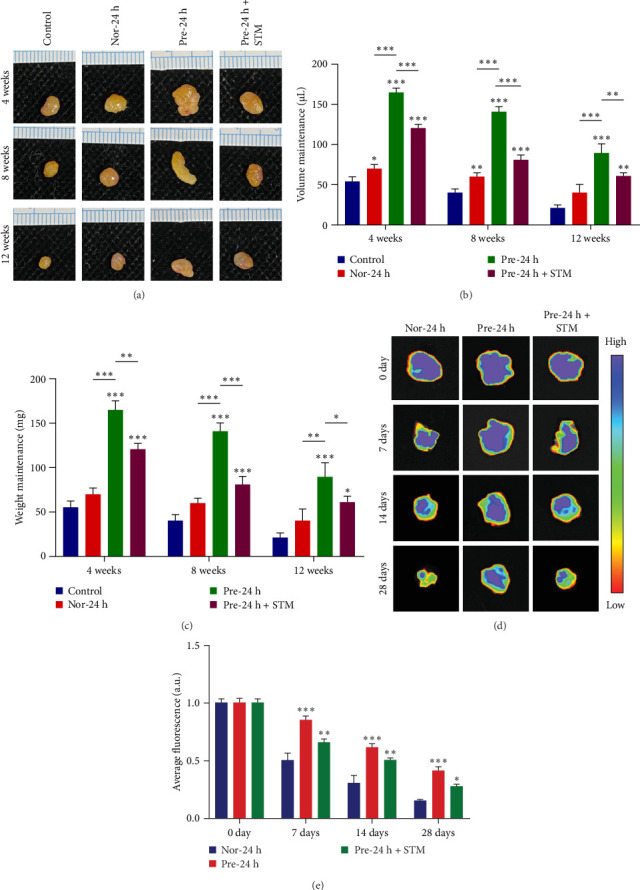
Hypoxia preconditioning ADSCs promote fat graft retention. (A) Gross results at 4, 8, and 12 weeks after fat grafting. (B) Volume statistics of the fat grafts. (C) Weight statistics of fat grafts. (D) GFP-labeled ADSCs by an in vivo imaging system. (E) In vivo imaging fluorescence intensity statistics. Data are presented as mean ± SD. Statistical significance: *⁣*^*∗*^*p* < 0.05, *⁣*^*∗∗*^*p* < 0.01, and *⁣*^*∗∗∗*^*p* < 0.001.

In the above study, we further discussed the effects of ADSCs cultured under normoxic conditions and ADSCs pretreated with hypoxia, as well as ADSCs pretreated with hypoxia +METTL3 inhibitor, on the effects of fat grafting. Next, we validated this phenomenon in a BALB/c nude mouse fat grafting model. The experiment was divided into four groups: saline + adipose tissue (Control), normoxic ADSCs + adipose tissue (Nor-24 h), hypoxic preconditioned ADSCs + adipose tissue (Pre-24 h), and hypoxic preconditioned ADSCs + METTL3 inhibitor + adipose tissue (Pre-24 h+STM). We collected samples at weeks 4, 8, and 12 after transplantation to test the retention of transplanted fat. The results showed better fat retention in all other groups compared to the control group (Figure 5A). By measuring graft volume (Figure 5B) and weight (Figure 5C), we found that the volume and weight retention of the Nor-24 h group, Pre-24 h group, and Pre-24 h+STM treatment group were significantly higher than that of the control group at weeks 4, 8, and 12 (*p* < 0.05). In addition, there were also significant differences in fat volume and weight retention in the Pre-24-h group compared with the Nor-24-h group and the Pre-24-h+STM treatment group (*p* < 0.01). To further assess the survival of ADSCs after transplantation, we tracked GFP-labeled ADSCs by an in vivo imaging system. The results showed that the fluorescence signal intensity of the Pre-24-h group consistently maintained the highest level for 28 days after transplantation, and its signal values on days 14 and 28 were increased by 46% and 31% compared with those of the Nor-24-h group, respectively (*p* < 0.01), suggesting that hypoxia preconditioning significantly prolonged the in vivo survival time of ADSCs. In contrast, the signal intensity of the Pre-24 h+STM treatment group decreased by 18%–23% (*p*  < 0.05) compared with the Pre-24 h group (Figure 5D). Thus, we found that hypoxic preconditioning enhanced the effect of ADSCs on adjuvant fat grafting, whereas inhibition of METTL3 activity reduced the enhancing effect of hypoxic preconditioning on ADSCs.

**Figure 6:**
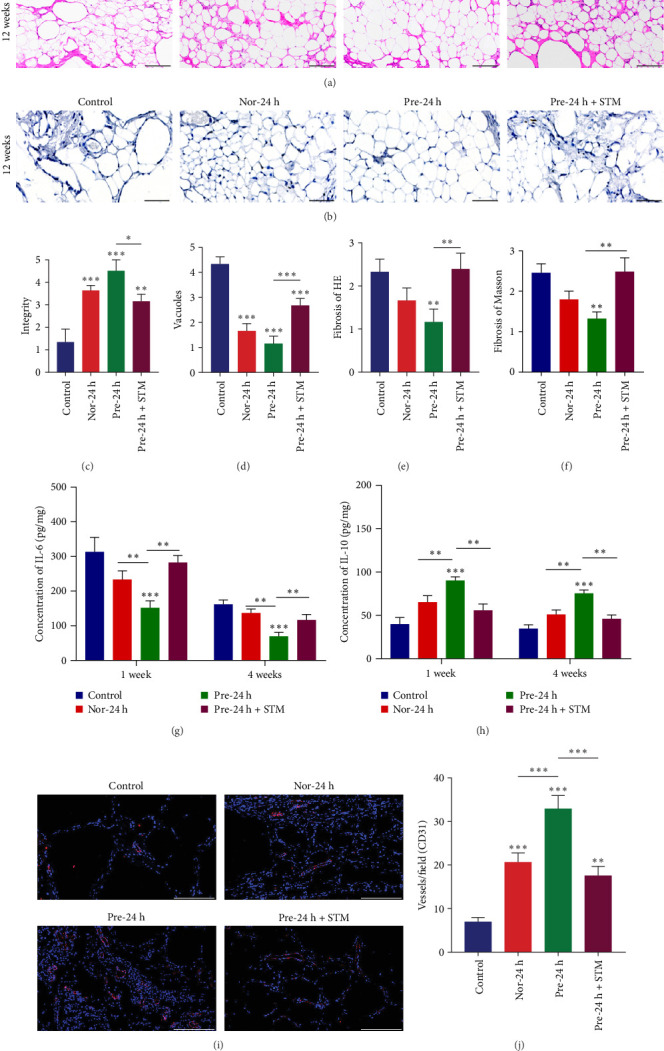
Histological examination and CD31 immunofluorescence staining of adipose tissue after grafting. (A) HE staining of adipose tissue (scale bar, 200 μm). (B) Masson staining of adipose tissue (scale bar, 200 μm) (C) Adipocyte integrity. (D) Degree of vesicularization. (E) HE staining for degree of fibrosis. (F) Mason staining for degree of fibrosis. (G) ELISA for the concentration of IL-6 (pg/mg). (H) ELISA for the concentration of IL-10 (pg/mg). (I) CD31 immunofluorescence staining (scale bar, 200 μm). (J) Vessel number counts. Data are presented as mean ± SD. Statistical significance: *⁣*^*∗*^*p*  < 0.05, *⁣*^*∗∗*^*p*  < 0.01, and *⁣*^*∗∗∗*^*p*  < 0.001.

To evaluate the long-term preservation effect of adipose grafting, we performed hematoxylin-eosin staining (HE staining) and Masson staining of adipose tissues after 12 weeks of grafting. HE staining showed that the morphology of adipose tissues was more intact in the Nor-24 h group and the Pre-24 h group after 12 weeks of grafting (Figure 6A). Masson staining further confirmed that the collagen fibers in the Pre-24 h group deposition was significantly less than that in the control and Pre-24h+STM groups, consistent with the fibrosis trend observed in HE staining (Figure 6B). We also assessed the adipocyte integrity, the degree of adipose tissue vesicularization and the degree of fibrosis by two pathologists using a 0–5 scale. The results showed that the adipocyte integrity was better in the Nor-24 h and Pre-24 h groups after 12 weeks of transplantation compared with the control and Pre-24h+STM groups; the adipocyte integrity in the Pre-24 h group was significantly higher than that in the Nor-24 h group (*p* < 0.05), and the difference was statistically significant (Figure 6C). After 12 weeks of transplantation, a large number of vesicles and cystic cavities were seen in the control and Pre-24h+STM groups (Figure 6A), which were caused by adipose tissue necrosis. In contrast, the degree of vesicle formation was significantly reduced in the Nor-24 h and Pre-24 h groups (*p* < 0.001, Figure 6D). In addition, HE staining showed thickening of fibrous connective tissue intervals in the control and Pre-24 h+STM groups, suggesting a higher degree of fibrosis. Masson staining further confirmed that the Pre-24 h group had the lowest degree of fibrosis, and the difference was significant compared with the remaining groups (*p* < 0.001, Figure 6E,F). It was thus evident that hypoxia-pretreated adipose stem cells effectively enhanced the preserved quality of fat grafted tissues and significantly inhibited the fibrosis process.

Given that inflammatory microenvironment regulation is a key factor affecting graft survival, we detected the levels of inflammatory factors at 1 week (acute phase) and 4 weeks (chronic phase) after transplantation by ELISA. The results demonstrated that during the acute inflammatory phase at 1 week posttransplantation, the Pre-24 h group exhibited significantly lower IL-6 levels compared to the control, Nor-24 h, and Pre-24 h+STM groups (*p* < 0.001), while showing markedly elevated IL-10 levels (*p* < 0.001, Figure 6G), indicating effective suppression of acute inflammatory responses. By the chronic phase at 4 weeks post-transplantation, the Pre-24 h group maintained the lowest IL-6 levels (*p*  < 0.001), and the highest IL-10 levels (*p*  < 0.001) among all groups (Figure 6H). These findings demonstrate that hypoxic preconditioning not only effectively alleviates early inflammatory injury but also persistently improves the transplanted tissue microenvironment, which shows high consistency with our previous observations of reduced fibrosis and enhanced graft survival.

In addition, we further investigated the promotional effect of hypoxia preconditioned ADSCs on fat graft angiogenesis. The results showed that the proportion of CD31-positive cells was significantly higher in the Nor-24 h, Pre-24 h and Pre-24 h+STM groups compared with the control group (*p*  < 0.01), and the number of blood vessels in the Pre-24 h group was significantly higher than that in the Nor-24 h and Pre-24 h+STM groups (*p* < 0.001, Figure 6I,J), and this group also exhibited the most significant anti-inflammatory effect (Figure 6G, H). This synergistic effect suggests that hypoxic preconditioning may jointly improve graft survival by attenuating the early inflammatory response and promoting vascularization.

## 4. Discussion

Surgeons have been using autologous fat as a filler for over a century. Although fat grafting holds great therapeutic promise, unpredictable fat necrosis and resorption have led to instability in long-term results. This has undoubtedly caused a great deal of distress for clinicians, and ways to improve retention after fat grafting have attracted extensive attention from researchers.

The present study provides insight into the effects of hypoxic preconditioning on ADSCs, particularly its potential to improve AFT retention. Previous studies have shown that a concentration of 2%–7% O_2_ better mimics the physiological hypoxic environment in which cells are exposed in vivo and is able to activate protective pathways, whereas less than 2% O_2_ is more prone to inducing pathological responses [35]. So 1% O_2_ was used to simulate extreme hypoxic conditions, while 5% O_2_ was used for hypoxic preconditioning conditions. Through a series of experiments, we observed that hypoxic preconditioning significantly increased the survival and antioxidant capacity of ADSCs and decreased the level of apoptosis, and these findings provide new perspectives for improving the success of AFT. Our results suggest that 5% O_2_ treatment for 24 h is the optimal hypoxic preconditioning condition for ADSCs, which enhances the antioxidant capacity, reduces ROS levels, and decreases apoptosis. This is related to the cytoprotective mechanisms activated by hypoxic preconditioning, which may include the enhancement of antioxidant enzyme activities, the enhancement of metabolic adaptations of cells, and the activation of anti-apoptotic signaling pathways.

As an important epigenetic regulation, m6A modification plays a role in a variety of cellular processes. Our study found that hypoxic preconditioning upregulated METTL3 expression in ADSCs, which in turn increased m6A modification levels. m6A modification mediated by METTL3 plays a key role in reducing apoptosis in ADSCs, which was confirmed by our experimental results after knocking down METTL3 expression by siRNA. In this study, we systematically explored the regulatory mechanism of m6A modification in ADSCs, verified the characterization of changes in modification levels, and screened key m6A modification molecules. It was found that hypoxic preconditioning reduced the level of apoptosis in ADSCs through METTL3-mediated m6A modification, revealing the regulatory role of this molecular pathway. These findings corroborate with previous studies [42, 43]. on the regulation of ADSCs function by m6A modification, further expanding the cognitive boundaries of the field, providing a new theoretical basis for an in-depth understanding of the mechanism of ADSCs survival under hypoxic conditions, and pointing out important molecular targets for subsequent studies.

Bcl-2, as an important anti-apoptotic protein, was found to be upregulated at its expression level after hypoxic preconditioning in this study, but the exact regulatory mechanism is not clear. Therefore, we further predicted the m6A modification sites of Bcl-2 mRNA based on the SRAMP website (Supporting Information: Figure S3A,B), identifying multiple high-confidence sites (motifs GGACU, GAACU, GGACA, GAACU, GGACC). Previous studies have shown that after completing intranuclear m6A modification, METTL3 can continue to bind transcripts. After translocation to the cytoplasm, METTL3 can interact with eIF3, possibly leading to the formation of a linkage between METTL3 at the mRNA 3′ UTR and the 5′ mRNA cap structure, which drives the mRNA into a loop. This looped structure allows the ribosome to be reloaded onto the transcript at the stop codon, directly activating the translation process [44]. Analysis of the GSE2460366 dataset using the RMBase v2.0 database further revealed that the m6A modifications of Bcl-2 mRNA were mainly enriched at the junction of the coding sequence (CDS) and the 3′UTR, with a particular concentration near the termination codon (Supporting Information: Figure S3C). Meanwhile, the interaction between Bcl-2 mRNA and METTL3 was analyzed by catRAPID database, and the two were predicted to have a strong binding region between nucleotide positions 300 and 600 (Supporting Information: Figure S3D). To verify the association of Bcl-2 mRNA with m6A modification and METTL3, we performed RIP and m6A methylated RIP (meRIP) experiments. The results showed that Bcl-2 mRNA was significantly enriched in the m6A-specific immunoprecipitation group compared with the IgG control group (Supporting Information: Figure S3E). Similarly, the METTL3-IP group showed a higher level of Bcl-2 mRNA enrichment than the IgG control group (Supporting Information: Figure S3F). Taken together, the combination of METTL3-mediated upregulation of m^6^A levels and upregulation of Bcl-2 protein expression in ADSCs after hypoxic preconditioning suggests that hypoxic preconditioning mediates m^6^A modification of the critical regions of Bcl-2 mRNA (especially at the CDS-3′UTR junction) via METTL3. On the one hand, it may enhance the stability of Bcl-2 mRNA, and on the other hand, it may directly activate its translational efficiency by promoting METTL3-eIF3 interaction and mRNA cyclization, ultimately leading to an increase in Bcl-2 protein expression, and thus reducing the apoptosis rate of ADSCs. This finding provides new insights into the molecular mechanism of m^6^A modification in the regulation of apoptosis and points out the direction for further research.

In a BALB/c nude mouse model, we verified the effect of hypoxia-pretreated ADSCs in promoting fat retention in AFT. The results showed that hypoxia-pretreated ADSCs significantly increased the volume and mass retention of fat grafts and improved the morphology and structure of adipose tissue. In addition, hypoxia-pretreated ADSCs showed the ability to promote neovascularization, which may be another important mechanism for improving fat graft retention.

In summary, the present study revealed the molecular mechanism by which hypoxic preconditioning improves survival and reduces apoptosis in ADSCs through METTL3-mediated m6A modification and validated the potential of hypoxic preconditioned ADSCs in improving AFT retention in an animal model. These findings provide new strategies to improve the clinical outcomes of fat grafting. Future studies will further explore the specific mechanism of action of m6A modification in ADSCs and its potential role in other types of stem cells and develop new approaches for clinical applications.

## 5. Conclusion

In this study, we investigated that METTL3-mediated m6A modification plays a key role in reducing apoptosis in ADSCs and screened that 5% O_2_ treatment for 24 h is the optimal hypoxic preconditioning condition for ADSCs, which enhances the antioxidant capacity, reduces apoptosis, and promotes neovascularization in ADSCs. Its potential in improving AFT retention was revealed, providing new molecular mechanisms and therapeutic targets for improving AFT retention. These findings provide new strategies for the clinical application of ADSCs and are expected to improve the long-term outcome of fat grafting.

## Data Availability

The data that supports the findings of this study are available in the Supporting Information of this article.
