# Supplementary material for: METTL3-Mediated m6A Modification Enhances the Function of Adipose-Derived Stem Cells Under Hypoxic Conditions Thereby Improving Fat Graft Retention
Source: Stem Cells Int. 2025 Nov 30;2025:5589397. doi: 10.1155/sci/5589397 (PMC12682458; doi:10.1155/sci/5589397)
Supplement: Supporting Information — Supporting figures S1, S2, and S3 are provided in the Supporting Information. [file 5589397.f1.pdf]

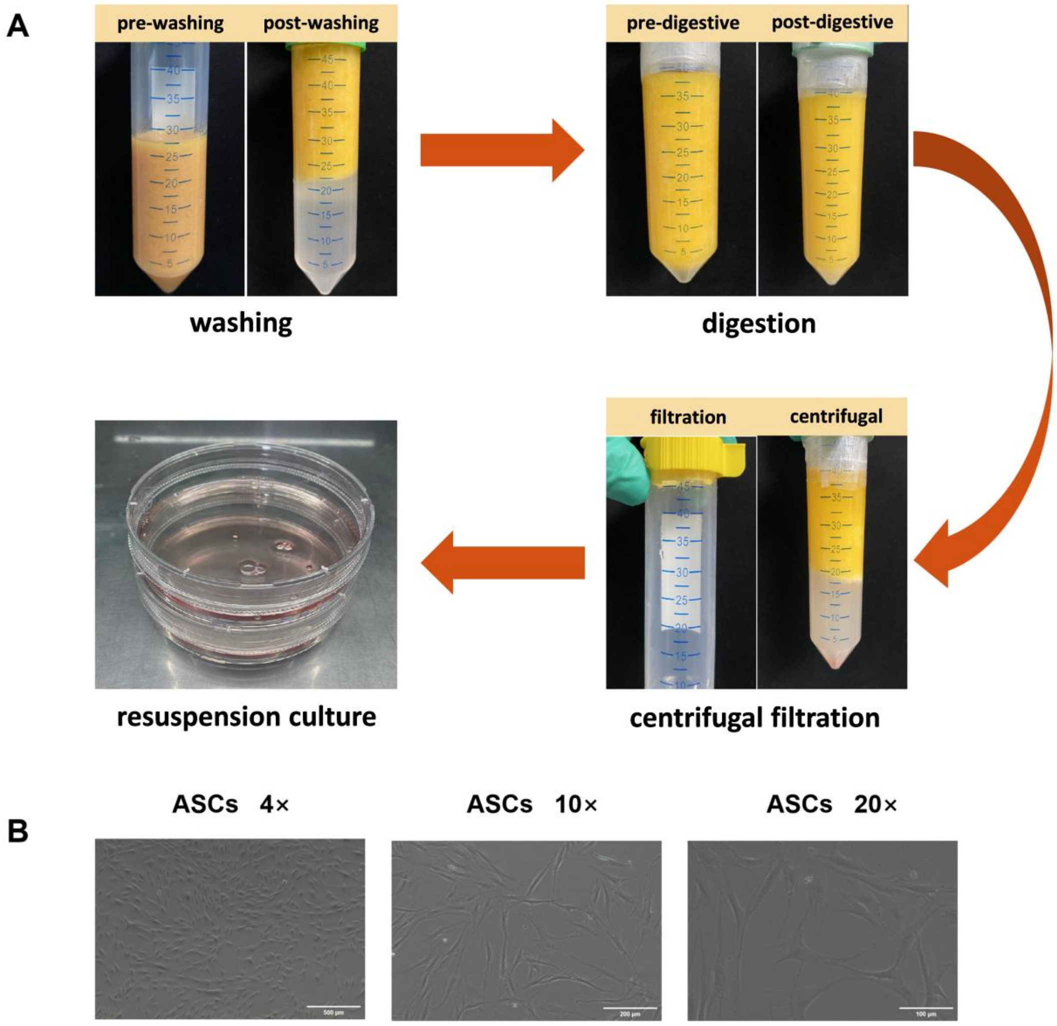

Supplementary figure 1. Primary Extraction of ADSCs and Morphological Observations of ADSCs.

(A) Flow chart of the extraction of ASCs. (B) Cell morphology of ASCs under 4×, 10×, and 20× light microscopy. (Scale bars, 500 μm, 200 μm, 100 μm)

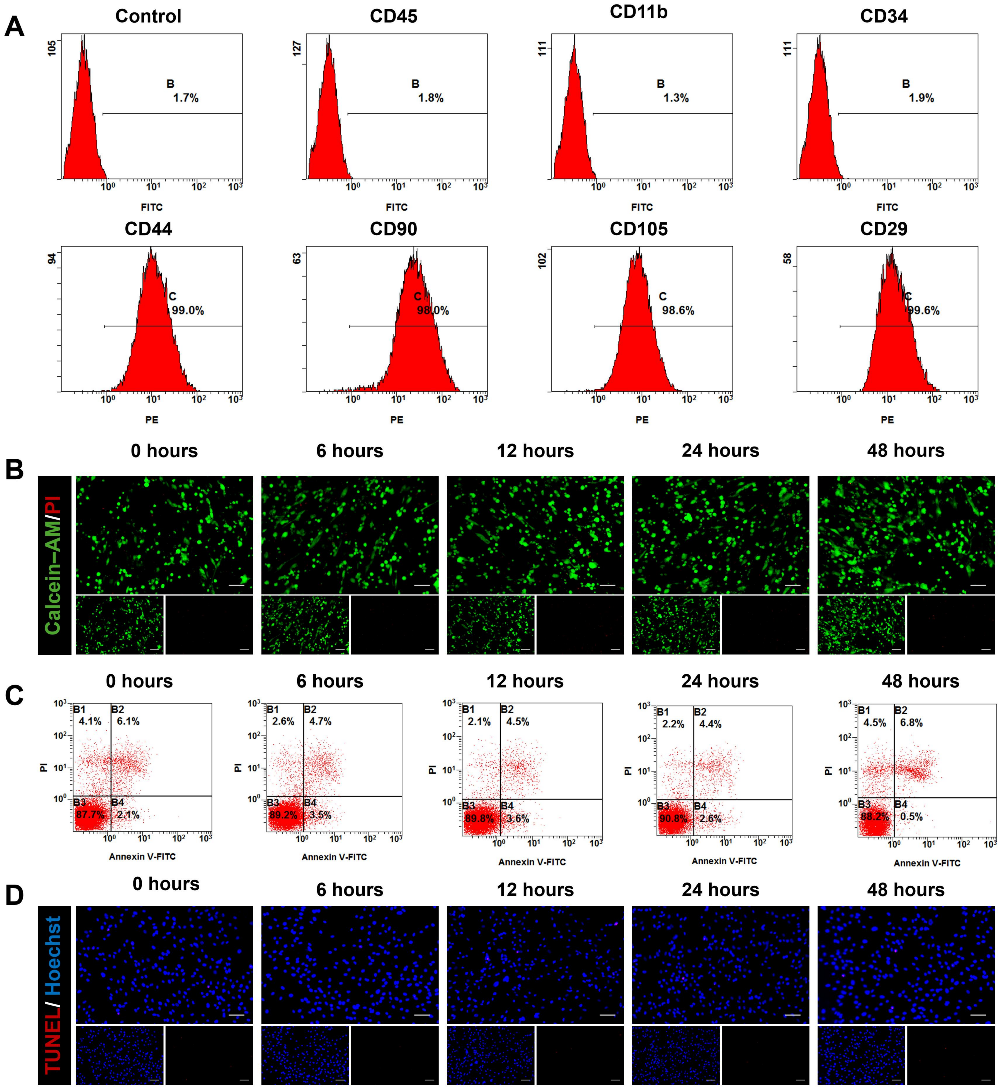

Supplementary figure 2. Flow cytometry identification and viability testing of ADSCs.

(A) Flow cytometry-based characterization of ASCs, which showed negative CD45 (1.8%), CD11b (1.3%), and CD34 (1.9%) and positive CD44 (99.0%), CD90 (98%), CD105 (98.6%), and CD29 (99.6%). (B) Calcein AM/PI assay for cell viability levels (Scale bar, 100 μm). (C) Flow cytometry was used to detect the apoptosis level. (D) TUNEL staining to detect the apoptosis level (Scale bar, 100 μm).

A

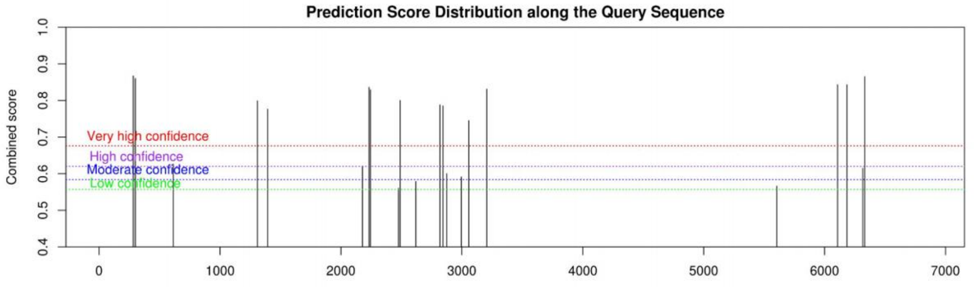

B

Position

#	Position	Sequence context	Structural context	Local structure visualization	Score(binary)	Score(knn)	Score(spectrum)	Score(combined)	Decision
1	283	GAGAA GGGGA AAACA<br>UCACA GGACU UCUGC<br>GAAUA CCGGA CUGAA	N/A	N/A	0.921	0.719	0.810	0.867	m^{6}A site (**Very high** confidence)
2	300	ACAGG ACUUC UGCGA<br>AUACC GGACU GAAAA<br>UUGUA AUUCA UCUGC	N/A	N/A	0.899	0.661	0.832	0.860	m^{6}A site (**Very high** confidence)
3	614	UACAA CAUCA CAGAG<br>GAAGU AGACU GAUAU<br>UAACA AUACU UACUA	N/A	N/A	0.671	0.59	0.570	0.626	m^{6}A site (**High** confidence)
4	1310	GAGCU CUUCA GGGAC<br>GGGGU GAACU GGGGG<br>AGGAU UGUGG CCUUC	N/A	N/A	0.829	0.453	0.802	0.799	m^{6}A site (**Very high** confidence)
5	1394	CGGGA GAUGU CGCCC<br>CUGGU GGACA ACAUC<br>GCCCU GUGGA UGACU	N/A	N/A	0.785	0.579	0.788	0.776	m^{6}A site (**Very high** confidence)
6	2178	GCCUC CCUGG CCUGA<br>AGAAG AGACU CUUUG<br>CAUAU GACUC ACAUG	N/A	N/A	0.675	0.676	0.540	0.621	m^{6}A site (**High** confidence)
7	2233	GGUGG GAGGA AAAGA<br>GUUGG GAACU UCAGA<br>UGGAC CUAGU ACCCA	N/A	N/A	0.84	0.519	0.870	0.836	m^{6}A site (**Very high** confidence)
8	2244	AAGAG UUGGG AACUU<br>CAGAU GGACC UAGUA<br>CCCAC UGAGA UUUCC	N/A	N/A	0.861	0.316	0.851	0.829	m^{6}A site (**Very high** confidence)

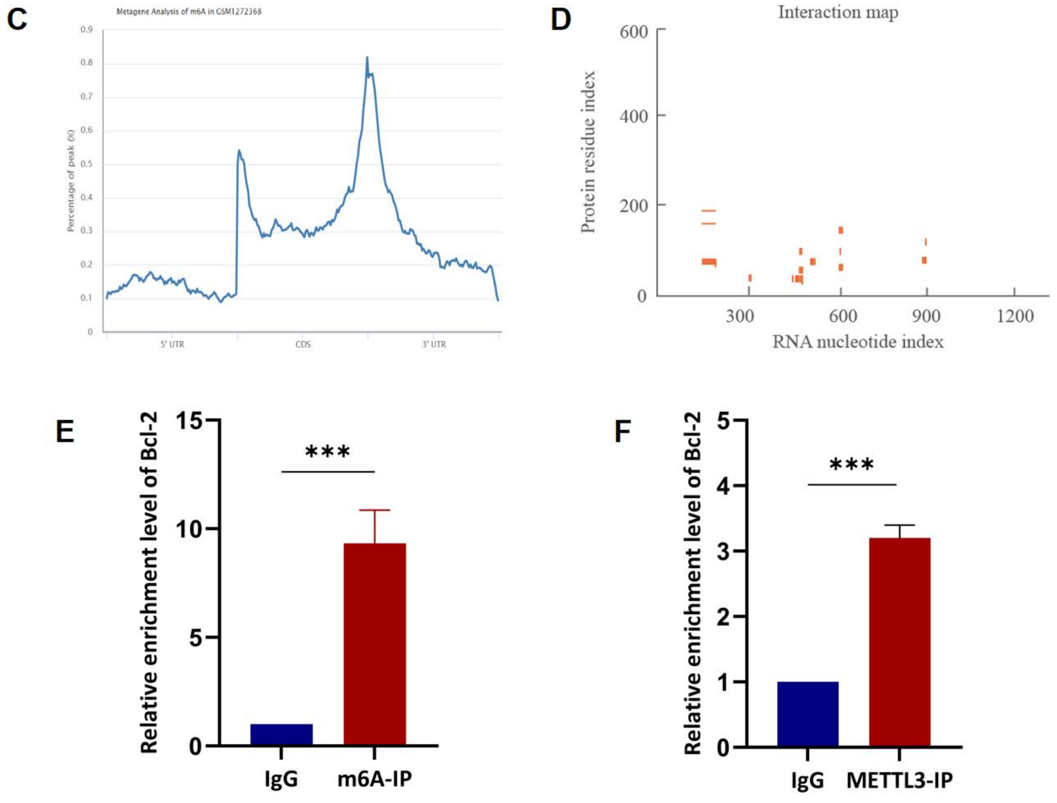

Supplementary figure 3. Prediction of m6A binding site of Bcl-2 mRNA.

(A) The m6A modification sites of Bcl-2 mRNA were predicted based on the

SRAMP website. (B) The prediction results showed that there are multiple high-confidence m6A binding sites on Bcl-2 mRNA. (C) Analysis of the GSE2460366 dataset using the RMBase v2.0 database. (D) The interaction between Bcl-2 mRNA and METTL3 was analyzed by catRAPID database. (E) meRIP shows the presence of m6A modification of Bcl-2 mRNA. (F) RIP shows that METTL3 interacts with Bcl-2 mRNA. Data are presented as mean ± SD. Statistical significance: * $p < 0.05$, ** $p < 0.01$, and *** $p < 0.001$.
